# Primary Bronchopulmonary Actinomycosis Masquerading as Lung Cancer: Apropos of Two Cases and Literature Review

**DOI:** 10.1155/2015/609637

**Published:** 2015-06-04

**Authors:** Stamatis Katsenos, Iosif Galinos, Panagiota Styliara, Nikoletta Galanopoulou, Konstantinos Psathakis

**Affiliations:** ^1^Department of Pneumonology, Army General Hospital of Athens, 138 Mesogion & Katehaki Avenue, 115 25 Athens, Greece; ^2^Department of Internal Medicine and Infectious Diseases, Army General Hospital of Athens, 138 Mesogion & Katehaki Avenue, 115 25 Athens, Greece

## Abstract

Actinomycosis is a rare and slowly progressive infectious disease that can affect a variety of organ systems including the lung. It is caused by filamentous Gram-positive anaerobic bacteria of the genus *Actinomyces*. Despite its rarity, pulmonary actinomycosis can involve lung parenchyma, bronchial structures, and chest wall. The disease can mimic lung malignancy given its nonspecific clinical and radiological presentation, thus posing a diagnostic dilemma to the attending physician. In this paper, we describe two patients with pulmonary actinomycosis mimicking bronchogenic carcinoma; the former presented with peripheral infiltrate and associated hilar/mediastinal lymphadenopathy and the latter presented with a foreign body-induced endobronchial mass. Clinical, imaging, diagnostic, and therapeutical aspects of the disease are discussed, demonstrating the paramount importance of the histological examination of lung tissue specimens in the confirmation of the infection given either its low culture yield or the limited use of new molecular diagnostic tools in routine clinical practice.

## 1. Introduction

Actinomycosis is a chronic, slowly progressive granulomatous disease caused by filamentous Gram-positive anaerobic or microaerophilic bacteria of the family Actinomycetaceae (genus* Actinomyces*) [1]. The pulmonary form accounts for 15% of all actinomycosis cases and it is thought to be caused primarily by the inhalation or aspiration of oropharyngeal or upper gastrointestinal materials [2]. It can occasionally occur after local spread of cervicofacial infection or hematogenous spread. The infection can involve lung parenchyma, central airways, pleura, mediastinum, and chest wall, thus causing several clinical complications such as bronchial obstruction, pleural empyema, chest wall swelling and fistulae, rib destruction, and superior vena cava obstruction. It is clinically seldom suspected at first presentation given its nonspecific clinical and radiological appearance that could easily represent other lung diseases, such as neoplastic disease, tuberculosis, pneumonia, and pulmonary abscesses. But, in essence, the most frequently initial suspected diagnosis is lung carcinoma, thus rendering the diagnosis of pulmonary actinomycosis more problematic [3]. Herein, we report two biopsy-proven cases of pulmonary and endobronchial actinomycosis presenting with clinical and radiological features suggestive of lung cancer.

## 2. Case Reports

### 2.1. Case 1

**Figure 1:**
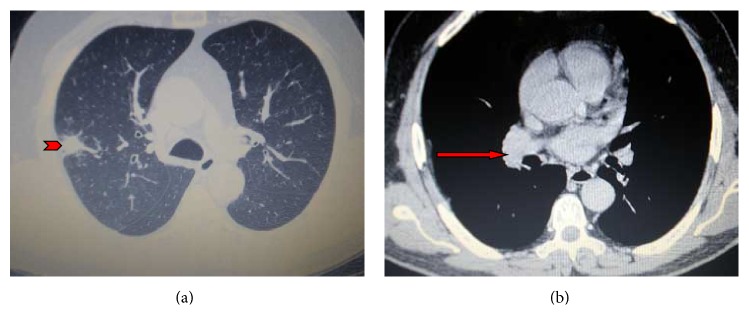
(a and b) Contrast-enhanced chest computed tomography showing a nodular opacity in the posterior segment of the right upper lobe (arrowhead) accompanied by mild ipsilateral pleural thickening and bilateral mediastinal lymphadenopathy (red arrow).

**Figure 2:**
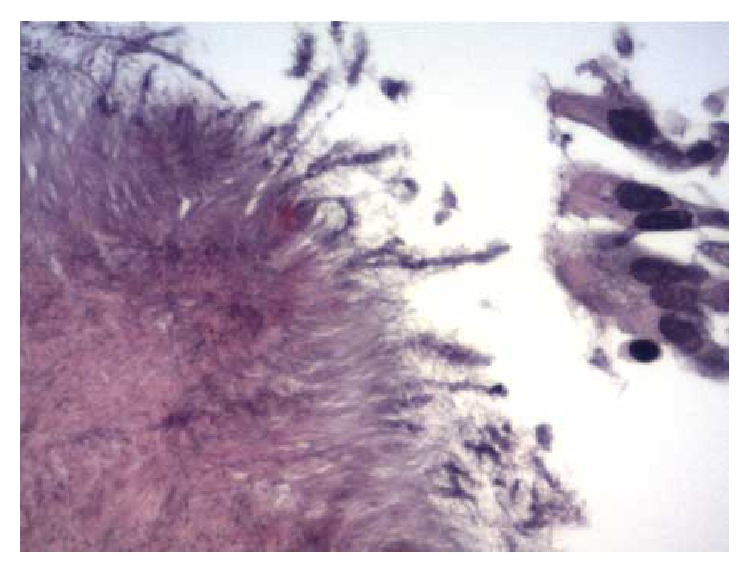
Bronchoscopic biopsy specimen demonstrating colonies of organisms with radiating eosinophilic terminal clubs on staining with haematoxylin-eosin (original magnification ×400).

A 67-year-old male, heavy smoker (40 packs/year), was admitted to our department presenting with intermittent hemoptysis one week before his admission. His medical history included a controllable arterial hypertension and a localized pleural thickening at the right hemithorax as an inactive residuum resulting from a lower respiratory tract infection that occurred 7 years ago. Chest examination was unremarkable whereas chest radiograph showed an infiltrate in the right upper lobe with associated mild pleural thickening. Further imaging evaluation with contrast-enhanced chest computed tomography revealed a nodular opacity in the posterior segment of the right upper lobe accompanied by mild ipsilateral pleural thickening and bilateral mediastinal lymphadenopathy (Figures 1(a) and 1(b)). A convex-probe endobronchial ultrasound (BF-UC160F-OL8, Olympus, Tokyo, Japan) was subsequently performed showing an enlarged right hilar lymph node 2.5 cm in diameter, bilateral subcarinal lymphadenopathy, and enlarged lymph nodes in the aortopulmonary window. Microbiologic examination of samples obtained by bronchial washings and BAL for pathogens was negative. EBUS-guided transbronchial needle aspiration (TBNA) was negative for malignancy. However, radial EBUS-guided transbronchial biopsies from the peripheral lesion showed Gram-positive filamentous branching bacteria and sulfur granules indicative of actinomycosis (Figure 2). The patient was directly given 24 million units of penicillin per day for 3 weeks followed by amoxicillin 2 gr daily for a total of six months, thus responding satisfactorily in this regimen in terms of clinical picture and imaging features. The disease has been completely regressed, as it is proven by the total resolution of the pulmonary infiltrate and mediastinal lymphadenopathy on a new CT scan.

### 2.2. Case 2

**Figure 3:**
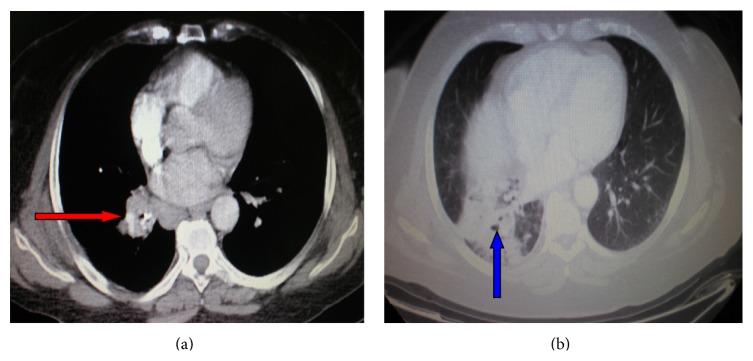
(a and b) A contrast-enhanced computed tomography of the thorax revealing a right hilar mass (red arrow) compressing the bronchus intermedius with accompanying dense airspace opacification of right lower lobe (blue arrow) and atelectasis.

**Figure 4:**
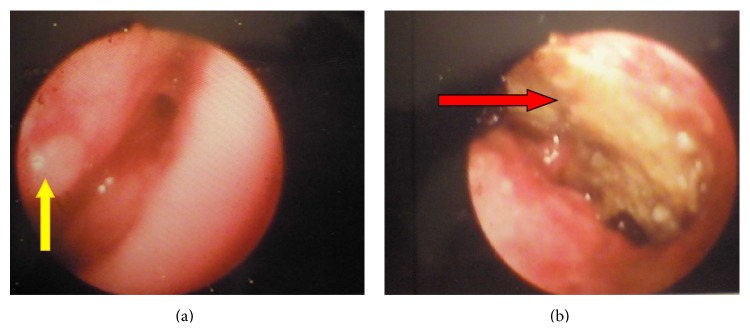
(a and b) Bronchoscopy showing a soft granulation tissue mass (yellow arrow) and an impacted foreign body (red arrow) occluding completely the right bronchus intermedius.

**Figure 5:**
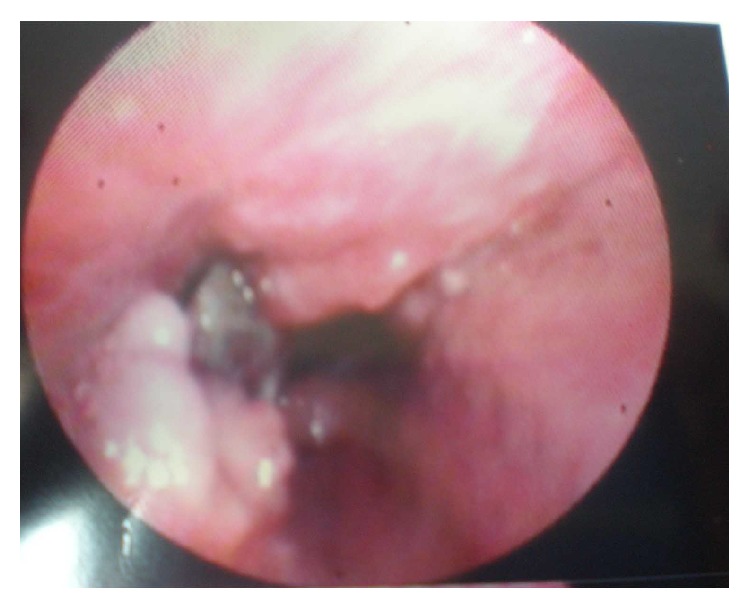
Repeat bronchoscopy after three weeks of antibiotic treatment showing remarkable resolution of the inflammatory mass, thus facilitating foreign body extraction.

A 70-year-old female, nonsmoker, was referred to our department for evaluation of persistent nonproductive cough with associated malaise. She reported the onset of symptoms 10 years ago without seeking any medical assistance. Clinical chest examination revealed diminished breath sounds in the right middle and lower lung fields. A posteroanterior chest radiograph showed right lung volume reduction with associated ill-defined consolidation in the right lower lung field. A contrast-enhanced computed tomography of the thorax demonstrated a right hilar mass compressing the bronchus intermedius with accompanying dense airspace opacification of right lower lobe and atelectasis (Figures 3(a) and 3(b)). Significant mediastinal or hilar lymph node enlargement was not identified. Rigid bronchoscopy was subsequently performed under general anesthesia followed by the insertion of the fiber optic bronchoscope, which demonstrated a soft granulation tissue mass and an impacted foreign body occluding completely the right bronchus intermedius (Figures 4(a) and 4(b)). The bronchoscopic view was indicative of bronchogenic carcinoma. However, large biopsy specimens obtained by rigid forceps showed colonies of organisms with radiating eosinophilic terminal clubs on staining with haematoxylin-eosin. The histopathology result was compatible with endobronchial actinomycosis. Foreign body removal was initially impossible as it is completely encased in a bulky and bleeding granulation tissue mass. Thus, actinomycosis treatment initiation with 24 million units of penicillin per day for 3 weeks given that the patient was clinically stable was decided. Bronchoscopic reexamination after three weeks resulted in remarkable resolution of the inflammatory mass, thus facilitating foreign body extraction (medium-sized fish bone) and obviating the need for surgical interventions (Figure 5). The patient switched to amoxicillin 2 gr daily for a total of six months exhibiting complete recovery.

## 3. Discussion

Actinomycosis is a chronic suppurative infection caused by* Actinomyces* spp., which are facultative anaerobic, branching Gram-positive, acid-fast negative bacilli, belonging to the normal flora of the oropharynx and the gastrointestinal and urogenital tract [4]. Practically, any organ system of the human body can be affected; pulmonary form comprises 15% of all actinomycosis cases and it is primarily caused by aspiration of oropharyngeal or upper gastrointestinal materials, albeit it can sometimes occur after local spread of cervicofacial infection or hematogenous spread [2, 5].

The most important risk factors for the development of pulmonary actinomycosis include poor oropharyngeal hygiene, preexisting dental disease, and alcoholism. In addition, chronic lung disorders such as chronic obstructive lung disease, bronchiectasis, chronic mycobacterial disease, and aspergilloma are considered another risk factor because the damaged lung tissue forms an anaerobic milieu that favors the growth of this bacterium (actinomycete) [3]. Although pulmonary actinomycosis has been described in a high percentage of current or former smokers or patients with coexisting diabetes mellitus, recent data demonstrated a significant proportion of nearly 50% of actinomycosis patients without suffering from any comorbidity, thus suggesting that thoracic actinomycosis does not occur only in multimorbid patients. Antibiotic treatment with quinolone formulations prior to hospitalisation was documented in 50% of the patients and might be suspicious for the presence of rare pathogens, such as actinomycetes [6]. Moreover, there is recent and compelling evidence that severe immunosuppression is certainly not a predisposing factor for pulmonary actinomycosis. Despite its presence in severely immunosuppressed patients, the prevalence in this setting remains very low [1]. Extrapolating these data to the present descriptive study, the first patient was in good health before ailing from actinomycosis while the second one had extensive tooth decay.

Major presenting symptoms, although nonspecific, include cough, productive sputum, haemoptysis, chest pain, weight loss, and fever. This clinical appearance mimics a variety of other lung diseases, such as malignancy, tuberculosis, pneumonia, and pulmonary abscesses, making the diagnosis complicated since it is usually established after the lapse of several months. In addition, endobronchial actinomycosis may present with dyspnea due to airway obstruction. The most common radiological finding is consolidation followed by mediastinal or hilar lymph node enlargement, atelectasis, cavitation, ground-glass opacity, and pleural effusion [7].

The diagnosis of thoracic actinomycosis is substantially based on histopathological examination of lung tissue biopsies obtained by CT-guided transthoracic needle biopsy, bronchoscopic techniques, or even surgical resection. Sulfur granules are colonies of organisms that appear as round or oval basophilic masses with radiating eosinophilic terminal clubs on staining with haematoxylin-eosin, thus representing the typical histological feature of pulmonary actinomycosis [1, 2, 5]. Nevertheless, cultural identification should be carried out, albeit bacterial confirmation has been achieved in only a minority of cases. This is due to empirical antimicrobial pretreatment, overgrowth of associated bacteria, and the fastidious nature of actinomycetes. In the first case, the patient received a short course of macrolides before the definite definitive diagnosis; hence the actinomycetes were not detected in BAL. The presence of actinomycetes in sputum or bronchial secretions from patients with pulmonary comorbidities or lung cancer may cause diagnostic confusion, although it seems to mostly imply colonization of necrotized lung tissue rather than infection. Thus, primarily sterile specimens, such as lung tissue biopsies, obtained by procedures that minimize contamination risk should be attempted as the principal diagnostic step. In addition, newly developed molecular methods, such as PCR with 16S rRNA gene sequencing and mass spectrometry, seem to provide rapidly and accurately microbiological confirmation of the disease [8]. These techniques are not widely used in routine clinical practice because of their availability only in specialized laboratories and the paucity of clinical data on their sensitivity and specificity for clinical decision making.

The frequent suspicion of pulmonary malignancy usually prompts physicians to resort to diagnostic surgical procedures. Indeed, surgery was the most frequent diagnostic method in the past three decades. However, with the advent of novel and minimally invasive modalities, such as radiology-guided transthoracic puncture or bronchoscopic techniques (e.g., endobronchial ultrasound-guided transbronchial needle aspiration or biopsy for the evaluation of mediastinal lymphadenopathy and EBUS-guided transbronchial biopsy for peripheral lesions), the frequency of surgical procedures for diagnostic confirmation has dramatically declined to 2–15% [6]. Therefore, the recommended diagnostic algorithm includes either radiological imaging-guided percutaneous procedures or bronchoscopic techniques before a surgical approach should be considered, which may lead to considerable morbidity, discomfort, costs, and diagnostic delay.

Endobronchial actinomycosis is a rare form of the infection that can cause significant airway obstruction. It is associated with either the presence of broncholiths or foreign bodies [9, 10]. They contain endobronchial calcified material caused by erosion of calcified lymph nodes into the airway as a result of a granulomatous process. Broncholithiasis mostly antedates the pulmonary actinomycosis. Besides, foreign body aspiration has been associated with endobronchial actinomycosis. In this descriptive study, an aspirated sharp fish bone was the root cause for the development of endobronchial infectious disease.

The prognosis of this infection is good, as antibiotic treatment is generally curative. However, medical treatment failure can occur in patients in whom the advisable antibiotics are administered. The absence of an antibiotic response at 1 month is the only independent factor associated with a poor treatment outcome, according to a recently published study [11]. Intravenous penicillin at a dose of 18–24 million units daily for 2–6 weeks, followed by oral penicillin or amoxicillin for 6–12 months, is the antibiotic treatment of choice. Surgical treatment should be primarily performed in patients presenting with specific complications (e.g., massive hemoptysis and pleural empyema). Last but not least, measures to reduce existing risk factors, such as restoration of a poor dental status, management of aspiration syndromes, or therapy of impaired immune function, should be continuously taken and included in the overall management of the disease.

In conclusion, pulmonary actinomycosis is a rare and slowly progressing bacterial lung infection. Respiratory physicians should be alert to the fact that this infectious disease can mimic the malignancy process and perform all the appropriate diagnostic methods (e.g., CT-guided transthoracic biopsy and bronchoscopic techniques) to exclude it, even more invasive (e.g., surgery). It is generally a curable disease provided that it is early diagnosed.
